# Acetabular reconstruction with femoral head autograft in primary total hip arthroplasty through a direct anterior approach is a reliable option for patients with secondary osteoarthritis due to developmental dysplasia of the hip

**DOI:** 10.1007/s00402-021-04187-2

**Published:** 2021-09-28

**Authors:** Dominik Kaiser, Emanuel Ried, Patrick O. Zingg, Stefan Rahm

**Affiliations:** Department of Orthopedics, Balgrist University Hospital, Zurich, Switzerland

**Keywords:** Acetabular augmentation, Femoral head autograft, Developmental dysplasia of the hip, Direct anterior approach

## Abstract

**Background:**

Developmental dysplasia is challenging to treat with total hip arthroplasty via the direct anterior approach (DAA). Reconstructing the former anatomy while restoring the acetabular bone stock for future revisions in this young patient collective combined with the known advantages of the DAA would be desirable. The purpose of this study was to analyze the feasibility, radiographic outcome and clinical outcome of primary uncemented total hip arthroplasty with bulk femoral head autograft for acetabular augmentation through a DAA with a minimal follow-up of 12 months.

**Methods:**

A retrospective, consecutive series from March 2006 to March 2018 of 29 primary total hip arthroplasty with acetabular augmentation with bulk femoral head autograft through a direct anterior approach was identified. All complications, reoperations and failures were analyzed. Radiographic and clinical outcome was measured.

**Results:**

24 patients (29 hips) with a mean age of 43 (18–75) years and a mean follow-up of 35 months (12–137) were included. Surgical indication was secondary osteoarthritis for developmental dysplasia of the hip (Hartofilakidis Grade A (*n* = 19), B (*n* = 10)) in all cases. We noted no conversion of the approach, no dislocation and no acetabular loosening. The center of rotation was significantly distalized by a mean of 9 mm (0–23) and significantly medialized by a mean of 18 mm (6–29). The bone graft was fully integrated after 12 months in all cases.

**Conclusion:**

Acetabular reconstruction with femoral head autograft in primary THA through a direct anterior approach seems to be a reliable option for the treatment of secondary osteoarthritis in patients with DDH Hartofilakidis grade A and B. Prospective cohort studies with a large sample population and a long-term follow-up are necessary to confirm our findings.

## Introduction

Developmental dysplasia of the hip (DDH) predisposes to early secondary osteoarthritis of the hip [1]. Despite new born screening programs some cases are missed, treated incorrectly or insufficiently. These patients often develop secondary osteoarthritis needing a total hip arthroplasty (THA) at an average age of 53 years old [2]. Due to the typically anterolateral and superior acetabular deficiencies, an increased femoral antetorsion, decreased intramedullary canal size and either coxa vara or valga THA in a dysplastic hip is technically more challenging [3]. For acetabular reconstruction, different techniques are described ranging from autologous bone reconstruction, metal augments, reinforcement rings to cranial positioning of the acetabulum [4]. The anatomical reconstruction of the center of rotation (COR) in particular by filling the acetabular defect with a femoral head has several advantages. It has been shown that a medialization and distalization of the COR positively affects hip function [5–7] and has been associated with increased survival of THA [8–10]. Furthermore, it may possibly decrease the rate of aseptic loosening [11, 12], as differences of as little as 5 mm in superolateral displacement decreases abductor function and relevantly deteriorates the ratio of body weight moment arm to abductor moment arm [12, 13]. In THA with acetabular autologous bone wedge augmentation, the center of rotation can be perfectly placed more medially and distally. The bone stock is increased and revision surgery is potentially facilitated [14, 15]. The possibility of using a smaller acetabular cup simplifies anterior osseous coverage and allows normal anteversion. Last but not least, the femoral head is readily available and cheap as a means for acetabular augmentation.

Augmentation of the deficient acetabulum has been traditionally performed through an anterolateral, lateral or posterolateral approach with encouraging short-, mid- and long-term results [14, 16–19]. However, the direct anterior approach (DAA) is known for several advantages like the true internervous and intermuscular plane resulting in less muscle damage and quicker early rehabilitation [20], less postoperative pain and pain medication [21–23], improved early postoperative mobilization, shorter hospital length of stay [23, 24], a greater proportion of patients discharged home vs. a rehabilitation center [25] and improved postoperative as well as early functional outcomes [26–28].

This study was designed to retrospectively analyze the reliability, the clinical outcome, surgical results, complications with and without implant revision as well as radiographic parameters in all primary THA with acetabular augmentation with a bulk femoral head autograft through a DAA.

## Materials and methods

This study was approved by our ethical review board (KEK-ZH-2020-02193) and all participants gave written informed consent.

### Patients

This retrospective, consecutive case series was conducted entirely at the author's institution. The patients were selected from May 2006 to March 2018. Inclusion criteria were acetabular augmentation with femoral head autograft for primary total hip arthroplasty through DAA with an uncemented cup. Exclusion criteria were patients receiving a different kind of acetabular augmentation, approach or a cemented acetabular cup. A total of 88 primary total hip arthroplasty with acetabular augmentation by bulk femoral head autograft were performed in this time frame. 59 were performed through a posterior approach and thus excluded. The study group comprised of 29 primary THAs in 24 patients with a mean age of 43 years (18–75). All patients were available for a last follow-up after a mean of 35 months (12–137). Indication was secondary osteoarthritis due to developmental dysplasia of the hip in all cases.

**Fig. 1:**
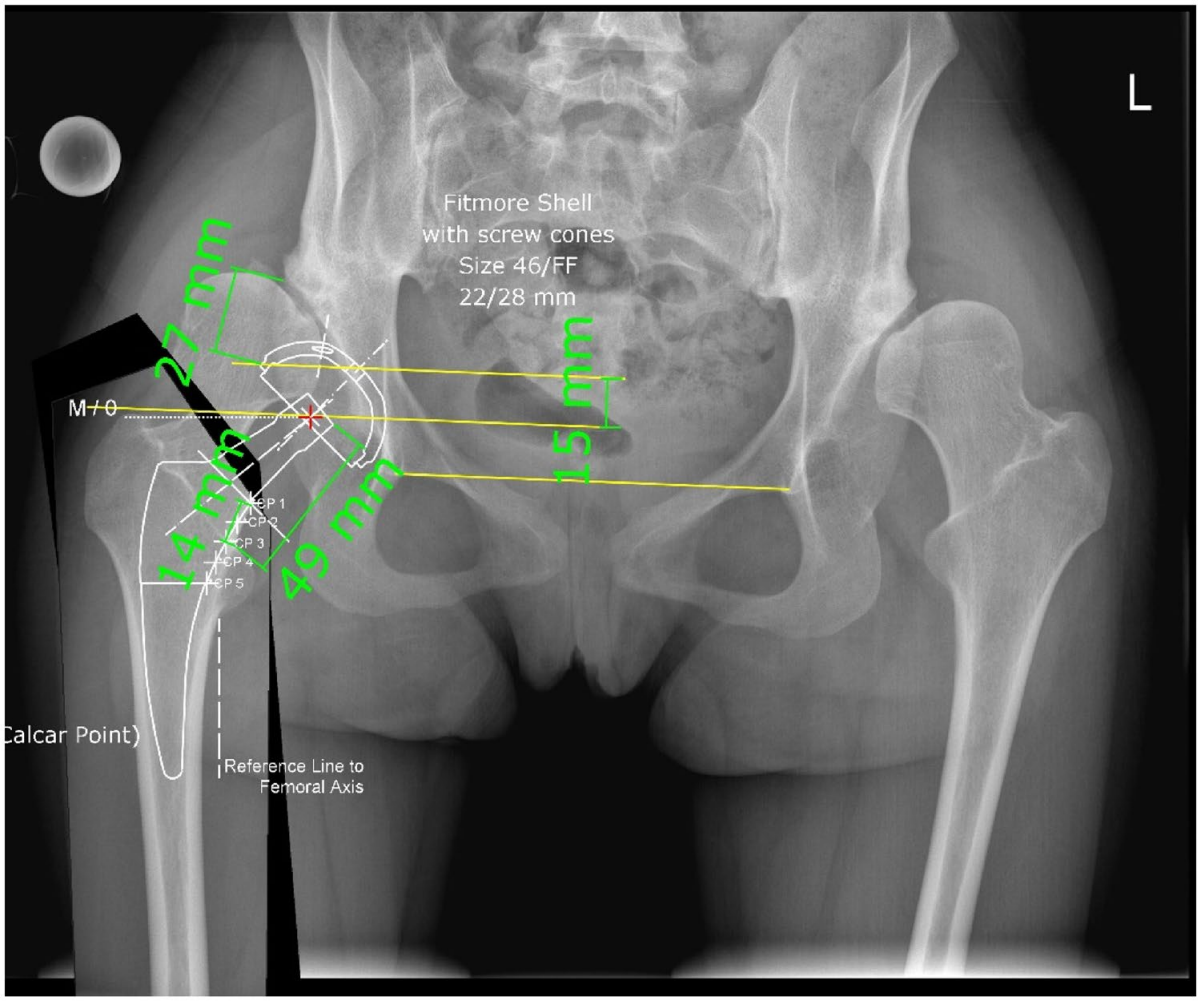
Preoperative THA template. Note the planned size of the bulk femoral head autograft wedge which is measured to have a width of 27 mm.

**Fig. 2:**
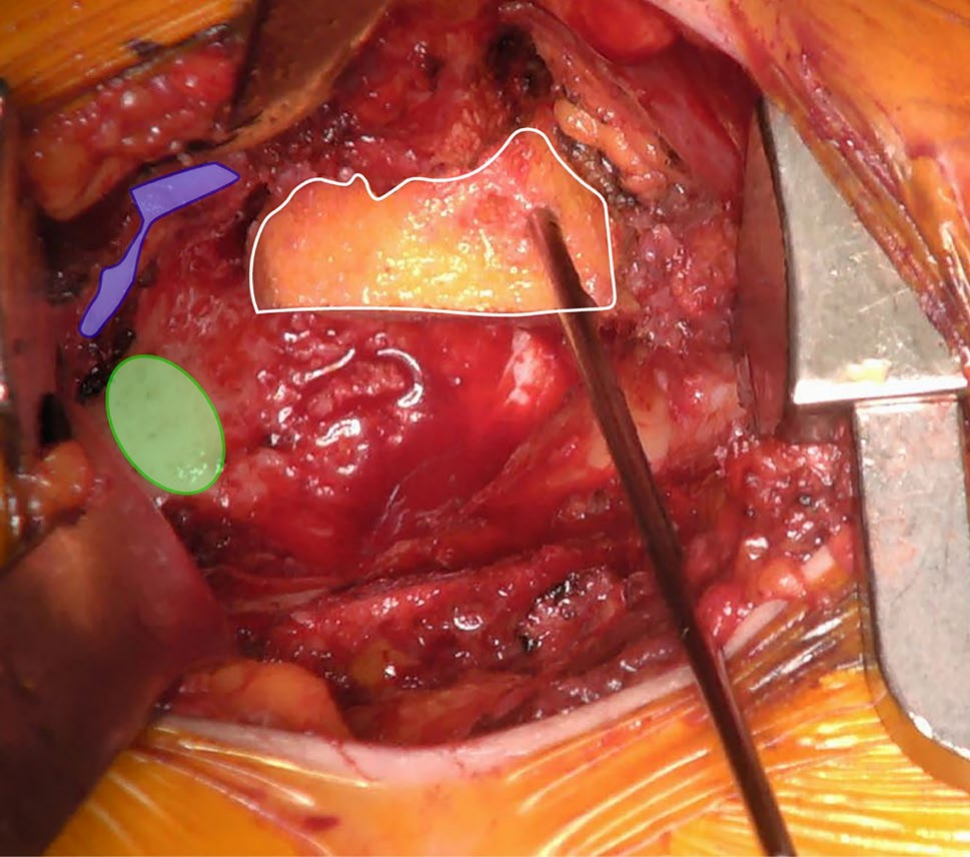
Intraoperative image of the preliminary fixation of the bulk femoral head autograft wedge (white framed) in the anterosuperior acetabulum. The wedge is preliminary fixed with a K-wire before definitive fixation with 3.5 mm fully-threaded cortical steel screws. Subsequently re- reaming can be performed typically starting with the smallest reamer. Highlighted are the anterior wall (purple) and the acetabular notch (green) (color figure online)

The surgical technique was performed in supine position with a traction table using Hueter interval in the anterior minimally invasive surgical approach technique, which is a modification of the DAA respecting a slightly lateral skin incision through the fasciae of the tensor fasciae latae to avoid problems with the lateral cutaneous femoris nerve [29, 30]. Acetabular reconstruction was performed using a wedge of the patient's femoral head. The size of the wedge was planned preoperatively by placing the acetabular cup at the desired position and then measuring the distance from the cup to the superolateral acetabular rim (Fig. 1). Remaining acetabular cartilage is removed with the reamer until pin-point bleeding is seen. The wedge is placed at the site of the greatest acetabular deficiency, mostly superolaterally. Preliminary fixation is obtained by a K-wire (Fig. 2). Definitive fixation of the femoral head graft was achieved with two sometimes three 3.5 mm fully threaded cortical steel screws under intraoperative fluoroscopy. Reaming of the acetabulum was started with a very small size and was gradually increased until press-fit was achieved and the planned acetabular position of the last reamer was controlled under fluoroscopy before the definitive cup was impacted. In all cases, either a Versafit cup (*n* = 12) (Medacta International, Castel San Pietro, Switzerland), a Fitmore cup (*n* = 17) and different press-fit or cemented stems were used including Quadra-H (*n* = 12) (Medacta International, Castel San Pietro, Switzerland), Fitmore (*n* = 14) (Zimmer Inc., Warsaw, IN, USA), Exafit (*n* = 2) (Zimmer Inc., Warsaw, IN, USA), CMK original (*n* = 1) (Zimmer Inc., Warsaw, IN, USA) depending on the patient's anatomy.

### Outcome measures

Demographic parameters, technical feasibility, clinical outcome measures including Western Ontario and McMaster Universities and Osteoarthritis Index [WOMAC, 0 = best, 10 = worst results] [31] and Harris Hip Score [HHS, 0 = worst, 100 = best results] [32], surgical results (surgical time, blood loss, acetabular cup size), complications with and without implant revision were recorded from our electronic patient's chart. Blood loss was calculated by subtracting the volume of the intraoperative irrigation fluid from the total volume in the collection tank. Radiographic parameters were obtained from the X-ray images.

### Radiography

**Fig. 3:**
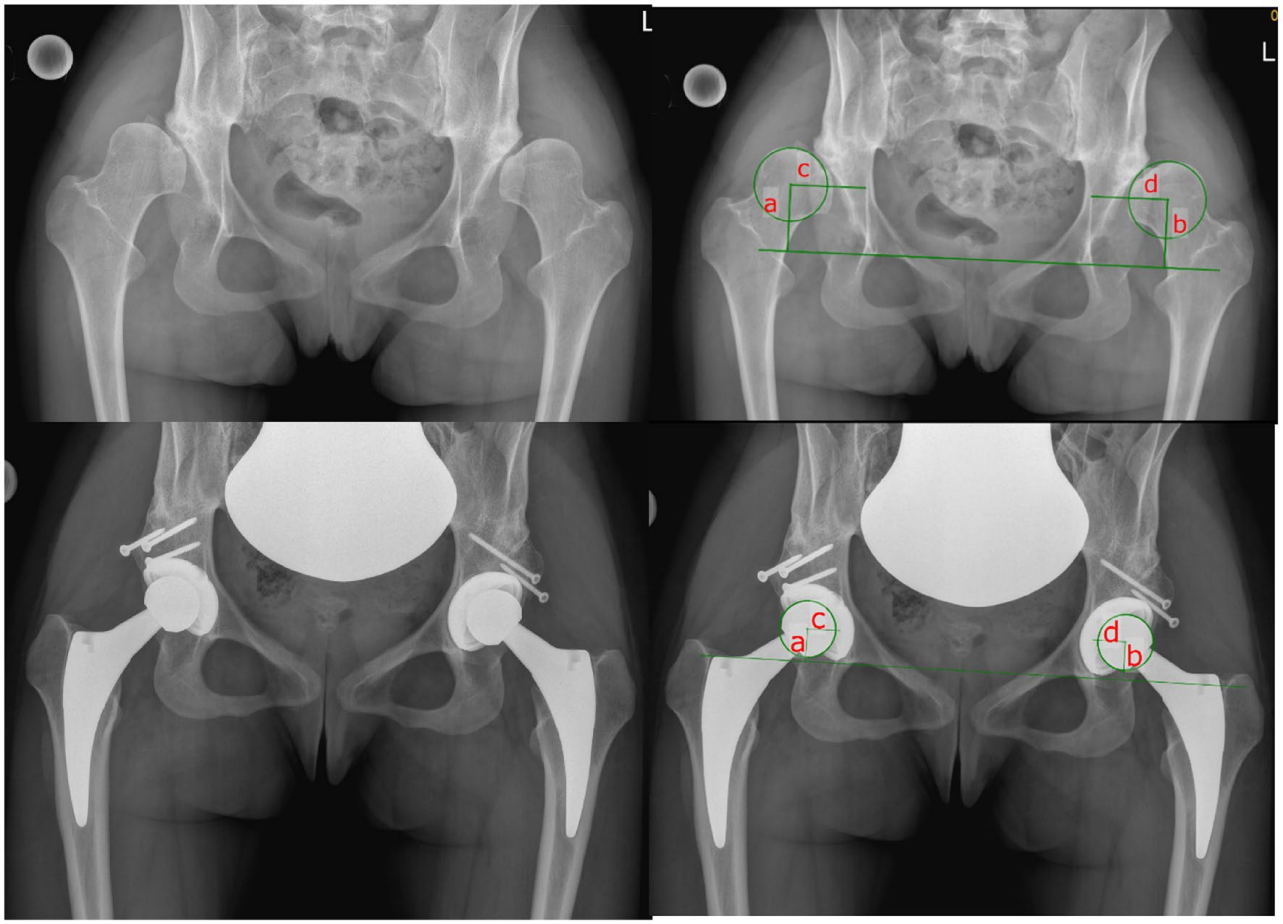
18 y/o patient with bilateral symptomatic secondary osteoarthritis due to DDH. Pre- and postoperatively after implantation of bilateral total hip replacement with autograft acetabular reconstruction (left side). Note the distinct distalization and medialization of the center of rotation. a and b depict the normal craniocaudal distance to the inter-teardrop line. The difference of the value of a in the upper right picture and the value of a in the lower right picture accounts for the change in the craniocaudal direction. c and d depict the mediolateral distance to the illioischial line parallel to the inter-teardrop line. The difference of the value c in the upper right picture to the value c in the lower right picture accounts for the change in the mediolateral distance. Note: In cases where the illioischial line is partly covered by the acetabular cup the illioischial line is extrapolated from the preoperative image and the continuation cranial and caudal of the cup

Pre- and postoperative standardized anteroposterior pelvic and axial X-rays were analyzed and were available for all 29 hips. The radiographs were analyzed for developmental dysplasia of the hip for intraarticular leg length discrepancy (comparing the lesser trochanter to a horizontal line defined by the two teardrop figures) and compared to the preoperative state, medialization and distalization of the center of rotation was compared pre- and postoperatively as well as to the preoperative planning (Fig. 3). Acetabular cup inclination/version was assessed using the technique of Lewinnek et al. [33].

The postoperative radiographs during follow-up visits were also analyzed for radiolucent lines in the acetabulum according to DeLee and Charnley I–III [34]. The bony integration of the autograft was assessed and inferred by the disappearance of the femoral head–host interface and when visible, the appearance of bridging trabeculae across this interface. The radiograph was evaluated for evidence of possible screw loosening.

### Statistical analysis

Statistical analysis was performed using the Mann–Whitney *U* test unless specified otherwise. Differences were considered to be statistically significant for P values < 0.05. Results of the Mann–Whitney U test are reported as means, range and associated *p*values if not stated otherwise.

## Results

The demographic information is depicted in Table 1.

**Table 1:** Demographic information

Number of patients	24
Number of hips	29
Side (right/ left)	14/15
Gender (female/ male)	17/7
Number of hips (female/ male)	19/10
Mean height (cm, range)	169 (156–188)
Mean age (years, range)	43 (18–75)
Mean BMI	24 (17–35)
Follow-up (months, range)	35 (12–137)

### Technical feasibility (n = 29)

Acetabular augmentation with femoral head autograft was feasible through a DAA in all cases. No conversion of the approach and no conversion of the acetabular augmentation was necessary to obtain the planned acetabular reconstruction.

### Clinical outcome (n = 29)

Clinical and surgical outcomes are depicted in Table 2.

**Table 2:** Clinical and surgical outcome

	Preoperative	Postoperative
Median WOMAC	5.5 (1.2–8.2)	0.5 (0–3.7)*
Median Harris Hip score	55 (13–83)	99 (80–100)*
Median blood loss (ml)	500 (200–1200)	
Mean femoral head size (mm)/Acetabular cup size	48 (42–53)	46 (44–50)*
Nr. of acetabular cup size 44/46/48/50	6/13/9/1	
Nr. of prosthetic head size 22/28 mm	1**/28	
Leg length discrepancy preoperative (mm)	10 (0–34)	4 (0–14)*
Autograft wedge size (planned/measured postoperatively) (mm)	18 (9–30)	18 (11–30)
Press-fit/ press-fit with screw augmentation	27/2	
Conversion of the approach***	0/29	

*A significant reduction was achieved postoperatively (*p* < 0.05) (Mann–Whitney *U* test)

**Dual mobility cup system

***From DAA to posterior

The mean WOMAC improved significantly from 5.5 (1.2–8.2) preoperatively to 0.87 (0–3.7) postoperatively (*p* < 0.001). The mean HHS Harris Hip score increased significantly from 55 (13–83) preoperatively to 97 (80–100) postoperatively (*p* < 0.001).

### Surgical results (n = 29)

Mean intraoperative blood loss was 564 ml (200–1200), with one patient needing postoperative blood transfusion. The mean planned acetabular wedge size was 18 mm (9–30) comparable to the postoperatively measured wedge size of 18 mm (11–30). A significantly smaller acetabular cup size was used with an average of 46 mm (44–50) compared to the preoperatively measured femoral head diameter of 48 mm (42–53, *p* = 0.026). Acetabular cups of the following sizes were used 44 mm: 6, 46 mm: 13, 48 mm: 9, 50 mm: 1. Sufficient acetabular press-fit was achieved in all patients. No patients needed a cage or a multi-hole revision cup.

Leg length discrepancy was significantly reduced from a preoperative mean of 10 mm (0–34) to a mean of 4 mm (0–14)) (*p* = 0.035), whereas one of the two patients with a postoperative difference of 14 mm was intentionally planned to have a remaining leg length difference of 14 mm from 35 mm. The patient feels balanced and is doing very well. The second patient with a leg length difference of 14 mm was due to a two-stage procedure for bilateral dysplasia and was planned and temporary.

### Complications (n = 29)

Overall there were five complications (17%) in four patients. We noted two nerve palsies of the lateral femoral cutaneous nerve (7%) which were both treated conservatively, one with pregabalin and one with a single perineural infiltration of local anesthetic. At the last follow-up, these patients had a WOMAC score of 1.1/0.1 and a HHS of 92 and 98. One deep venous thrombosis (3%) occurred despite prophylaxis with rivaroxaban 10 mg daily, treated conservatively without further medical consequences. We noted one superficial wound complication (3%) requiring wound debridement and closure of the skin without opening the fascia in an obese patient (BMI = 35 kg/m^2^). This occurred in the same patient who also developed a palsy of the lateral femoral cutaneous nerve treated by perineural infiltration (s. above). This patient's follow-up was 52 months and was uneventful. We noted one progressive acetabular osteolysis (3%) seen in an asymptomatic patient 60 months after index surgery potentially due to PE wear as the patient was running actively and a non-highly crosslinked liner was used. Twelve months after PE liner and femoral head change and 72 months after index surgery the patient remained asymptomatic and the radiological follow-up did not show progression of the acetabular osteolysis.

### Radiography (at 12 months after index surgery)

Radiographic information is summarized and depicted in Table 3. 19 hips were classified as Type A and 10 as Type B according to Hartofilakidis [35]. The mean planned size of the femoral autograft was 18 mm (9–30) and the postoperatively measured size was 18 mm (11–30). The maximal discrepancy of the planned and realized autograft was 6 mm in one case. No radiographic lucencies around the acetabular cup were seen 1 year after index surgery. Osseous anterior coverage of the acetabular cup as confirmed by a cross-table lateral radiograph was achieved in all patients. At the 1-year follow-up, all the bulk femoral head autografts were fully integrated in all patients and no loosening of the screws was seen. Adequate cup placement with a deviation of less than 3 mm to the planned COR in craniocaudal and mediolateral distance was seen in 80% of the patients. The center of rotation was significantly distalized by 9 mm (0–23, *p* < 0.0001) and significantly medialized by 18 mm (6–29, *p* < 0.0001). The achieved COR did not differ significantly from the planned COR in mediolateral direction (21 mm (15–32) vs. 22 mm (17–27)). The achieved COR on the other hand was significantly more distal then the planned COR (14 mm (5–20) vs. 17 mm (12–23), *p* = 0.04).

**Table 3:** Radiographic outcome

	Preoperatively	Postoperatively	1-year postoperatively
Acetabular inclination (°)	–	42 (30–51)	43 (33–53)
Acetabular anteversion (°)	–	20 (9–28)	21 (12–29)
Radiographic acetabular lucencies after 1 year	–	–	0
Radiographic femoral lucencies after 1 year	–	–	0
Osseous anterior coverage of the acetabular cup on axial X-ray	–	29/29	29/29
Hartofilakidis A/B	19/10	–	–
Autograft wedge size (mm)	18 (9–30) planned	18 (11–30) measured	–
Cranial distance from inter tear drop line to center of rotation preoperatively (mm)	24 (10–39)	14 (5–20)*	–
Lateral distance from illioischial line to center of rotation preoperatively (mm)	39 (27–55)	21 (15–32)*	–
Full integration of the autograft	–	–	29/29
Screw loosening	–	–	0/29

Values are depicted as mean and range

*A significant reduction was achieved postoperatively (*p* < 0.05) (Mann–Whitney *U* test)

## Discussion

The aim of this present study was to analyze the reliability, the clinical outcome, surgical results, complications with and without implant revision as well as radiographic parameters in primary THA with acetabular augmentation with a bulk femoral head autograft through a DAA. In our cohort, we were able to successfully perform acetabular augmentation and place an uncemented cup through the DAA in all cases without conversion of the approach. Thus, all the patients could benefit from the advantages of the DAA such as quicker rehabilitation, less postoperative pain, less pain medication and a shorter hospital length of stay as well as increased early functional outcomes [20–28]. While the muscle damage seen in this subgroup of patients (DDH) is greater than in less complex cases, this muscle damage especially seen in the obturator internus does not affect clinical outcome [36]. In addition to the advantages of the DAA discussed in the introduction, the supine position of the patient allows intraoperative fluoroscopy to verify the acetabular position not only regarding inclination and anteversion but also regarding the medialization and distalization of the COR. An average intended medialization of 18 mm and distalization of 9 mm of the COR was achieved [12, 13], increasing the lever arm and the pretension of the hip abductors, theoretically increasing hip function [6, 7] and potentially increasing longevity of the THA [8–12, 37]. The autograft was fully integrated after 1 year increasing the bone stock in these young patients potentially facilitating revision surgery. At last follow-up, the screws did not show any signs of loosening. No compromises had to be made regarding the anteversion of the cup (Table 3), despite the naturally shallow acetabulum and anterior wall deficiency typically seen in this patient collective. In our opinion, this is a clear advantage of using an autograft instead of using a larger acetabular cup, which is either implanted with clearly more anteversion or remains partially uncovered potentially leading to iliopsoas impingement [38]. As an alternative metallic foam augments could be used. In our opinion, the benefit of increasing the bone stock using an autograft is obvious and apart from the technical challenge and some fluoroscopy exposure there are no disadvantages. In addition, autografts are substantially cheaper than metallic foam augments.

After a mean follow-up of 35 months (12–137), no acetabular loosening was seen and no other acetabular cup was revised. In our series, we did not see any dislocation after a mean follow-up of 35 months (12–137), albeit a standard 28 mm prosthetic head was used in 28 cases and a DM cup was implanted in one patient due to the advanced age. This is less than the dislocation rate of approximately 3% described in the literature [39]. Achieving press-fit in the reconstructed acetabulum was possible in all cases; however, an additional screw fixation was deemed necessary in two cases.

An additional advantage of femoral head autografts is the increased pelvic bone stock which may facilitate revision surgery in the long term [15]. In the patients with a follow-up of more than 5 years (*n* = 7), the bone graft did not show radiological signs of resorption and remained fully integrated.

Uncemented acetabular components with femoral autografts for acetabular reconstruction in DDH have shown good short- and long-term results performed through anterolateral, lateral and posterolateral approaches [17–19, 40, 41].

The results presented in this study with THA performed through a direct anterior approach are comparable to the literature for femoral head autograft augmentation for DDH and acetabular segmental defects through other approaches. Zlatic et al. reported no acetabular loosening and three dislocations (5%) after a mean follow-up of 45 months in 61 patients and showed a full integration of the bone graft in all patients [19]. Yamaguchi et al. reported two (11%) acetabular loosening and no dislocations after a mean follow-up of 3.3 years in 18 hips and attributed the high rate of acetabular loosening to a lateral insertion of the acetabular component; a known risk factor [42]. This rate is higher than our 0% and is most likely explained by the high rate of severe dysplasia in their patient collective comprising of 55% Crowe type IV and possibly the slightly longer mean follow-up [43]. The bone grafts showed full integration in all 18 patients. DeWal et al. [40] reported no acetabular loosening in primary THA with 1 (7.7%) acetabular cup showing a radiolucency in all Charnley zones in a patient collective of 15 patients with a mean follow-up of 7.7 years. All grafts were fully incorporated without evidence of resorption. This study, however, can only be compared to ours to a limited extent as different indications were included, the most frequent indication, however, being DDH in seven cases (46.7%). Spangehl et al. reported 4 (9%) acetabular revisions, whereas only 1 (2%) was due to acetabular loosening and no dislocations in 44 patients after a mean follow-up of 7.5 years. 43 of 44 bone grafts showed no radiographic evidence of resorption [16].

There are limitations to the direct anterior approach in our hands. When a larger extension of the femur is necessary due to a higher degree of dysplasia, it is not possible to palpate the tension of the sciatic nerve through this approach. A femoral shortening osteotomy performed through a lateral subvastus approach would have to be done through a new incision. A distal extension of the direct anterior approach to perform a femoral shortening osteotomy poses a great risk for neurovascular structures supplying the quadriceps muscle [44]. We acknowledge that other authors perform THA for higher grade dysplasia as well with satisfactory results; however, we limit the indication to DDH grade A and B according to Hartofilakidis for the above-mentioned reasons. In higher grade dysplasia, we perform primary THA through a posterior approach to directly control the tension of the sciatic nerve and perform a femoral shortening osteotomy when necessary.

The present study has several limitations, including its retrospective design, relatively small cohort size, heterogeneity of implant models and the short minimal follow-up of 12 months [mean follow-up 35 months (12–137)]. However, there were no patients lost to follow-up and we do not expect any major changes in the results in the next 2–4 years as the acetabular cup was stable and the autograft is fully bony integrated in all patients. Therefore, this series shows a good and true validity and is of informative value. Patients with secondary osteoarthritis due to DDH benefit from the advantages of an anatomic placement of the COR and the DAA collectively.

## Conclusion

Acetabular reconstruction with femoral head autograft in primary THA through a direct anterior approach seems to be a reliable option for the treatment of secondary osteoarthritis in patients with DDH Hartofilakidis grade A and B. Prospective cohort studies with a large sample population and a long-term follow-up are necessary to confirm our findings.
